# Using temporal recalibration to improve the calibration of risk prediction models in competing risk settings when there are trends in survival over time

**DOI:** 10.1002/sim.9898

**Published:** 2023-09-13

**Authors:** Sarah Booth, Sarwar I. Mozumder, Lucinda Archer, Joie Ensor, Richard D. Riley, Paul C. Lambert, Mark J. Rutherford

**Affiliations:** ^1^ Biostatistics Research Group, Department of Population Health Sciences University of Leicester Leicester UK; ^2^ Oncology Biometrics Statistical Innovation, AstraZeneca Cambridge UK; ^3^ Institute of Applied Health Research, College of Medical and Dental Sciences University of Birmingham Birmingham UK; ^4^ Department of Medical Epidemiology and Biostatistics Karolinska Institutet Stockholm Sweden

**Keywords:** calibration, competing risks, prognostic models, risk prediction, temporal recalibration

## Abstract

We have previously proposed temporal recalibration to account for trends in survival over time to improve the calibration of predictions from prognostic models for new patients. This involves first estimating the predictor effects using data from all individuals (full dataset) and then re‐estimating the baseline using a subset of the most recent data whilst constraining the predictor effects to remain the same. In this article, we demonstrate how temporal recalibration can be applied in competing risk settings by recalibrating each cause‐specific (or subdistribution) hazard model separately. We illustrate this using an example of colon cancer survival with data from the Surveillance Epidemiology and End Results (SEER) program. Data from patients diagnosed in 1995–2004 were used to fit two models for deaths due to colon cancer and other causes respectively. We discuss considerations that need to be made in order to apply temporal recalibration such as the choice of data used in the recalibration step. We also demonstrate how to assess the calibration of these models in new data for patients diagnosed subsequently in 2005. Comparison was made to a standard analysis (when improvements over time are not taken into account) and a period analysis which is similar to temporal recalibration but differs in the data used to estimate the predictor effects. The 10‐year calibration plots demonstrated that using the standard approach over‐estimated the risk of death due to colon cancer and the total risk of death and that calibration was improved using temporal recalibration or period analysis.

## 1 INTRODUCTION

Developing prognostic models to produce long‐term survival or risk predictions relies on the inclusion of patients diagnosed many years ago in order to have sufficient follow‐up time to estimate the hazard rates, at for example, 10 years. However, if survival outcomes improve over time, and this is not accounted for during prognostic model development, then it can lead to out‐dated risk predictions for new patients that overestimate the risk of death from different causes as well as the total risk of death. Previous external validation studies of risk prediction models have identified this type of calibration drift and stress the importance of monitoring model performance over time^1^, ^2^, ^3^, ^4^, ^5^ and adjusting for miscalibration accordingly.^6^, ^7^, ^8^

In previous work, we proposed temporal recalibration as an approach to account for improvements in survival over time that can be applied at the model development stage and does not require any additional data.^9^ It is a two‐step process where the model is first developed on the full dataset to estimate the predictor effects. The model is then recalibrated by re‐estimating the (log cumulative) baseline hazard in a recent subsample of the data to update the baseline survival whilst constraining the predictor effects to remain the same as in the original model. This subsample is defined using a period window and the use of delayed entry allows improvements in baseline survival over time to be captured.

An alternative approach is to use period analysis^10^ to develop the prognostic model. Whilst in temporal recalibration the predictor effects are estimated using the full dataset, period analysis involves using just the subsample of data to estimate both the predictor effects and the baseline. This may lead to overfitting in derivation datasets with small numbers of participants or when the event of interest is rare.

Improvements in survival outcomes following a diagnosis of cancer have been widely reported over the past 20 years.^11^, ^12^, ^13^, ^14^ Using this knowledge during model development may lead to better calibrated risk predictions for new patients. We demonstrated this previously with a prognostic model for colon cancer patients, showing how temporal recalibration or period analysis can be used to account for trends in survival and lead to a prognostic model that produces better calibrated cause‐specific survival predictions in comparison to using the standard method where these temporal trends are ignored.^9^

A further issue is that many prognostic model development situations involve competing risks, which should be accounted for, as if ignored this also leads to risk predictions that are too high.^15^, ^16^ For example, when predicting the risk of death due to cancer, the competing risk of death due to other causes must be taken into account otherwise the predicted risk of death from cancer will be too high. In clinical practice, it may also be useful to understand not only a patient's risk of death from cancer but also their risk of death from other causes as this may affect treatment decisions,^16^ particularly for patients with comorbidities.

Established risk prediction models for cancer in England include PREDICT Breast,^17^ PREDICT Prostate,^18^ and QCancer Colorectal Survival.^19^ These models produce long‐term risk predictions that account for competing events by fitting a cause‐specific hazard model for death due to the cancer of interest and a second model for death due to other causes. However, how to also consider the issue of changes in survival over time remains unaddressed.

In this article, we demonstrate how temporal recalibration can be extended into a competing risk setting, to allow for prognostic model development in situations with both trends in survival and competing risks. We focus on the cause‐specific setting and show how to temporally recalibrate each of the cause‐specific hazard models separately, which can then be combined to produce the risk predictions. An alternative analysis using the Fine and Gray approach is included in Appendix A.7. We illustrate the approach using an example of survival following a diagnosis of colon cancer, and provide further applications for lung and breast cancer in Appendices A.5 and A.6.

## 2 METHODS

### 2.1 Calculation of risk predictions

The key estimands of interest for prognostic models in competing risk settings are the cause‐specific cumulative incidence functions (CIFs), $F_{k}\left(t|x_{i}\right)$. These give the probability of failure (risk) due to cause k, by time point t, for an individual with covariate pattern $x_{i}$, whilst accounting for the competing events. In the presence of competing risks, prognostic models can either be developed on the cause‐specific hazard scale or alternatively the effects of predictors can be modelled directly on the CIFs using models on the subdistribution hazards scale for example, with Fine and Gray models.^20^ Here we focus on the cause‐specific setting but further discussion surrounding Fine and Gray models can be found in Section 2.2.

The cause‐specific CIF for cause $k,F_{k}\left(t|x_{i}\right)$, can be defined as follows:

(1) $$F_{k}\left(t|x_{i}\right)=\int_{0}^{t}S\left(u|x_{i}\right)\;h_{k}\left(u|x_{i}\right)\;\mathrm{du},$$

which depends on the cause‐specific hazard function $h_{k}\left(t|x_{i}\right)$ and the all‐cause survival function $S\left(t|x_{i}\right).\;$The all‐cause survival function is the product of the k cause‐specific survival functions $S_{k}\left(t|x_{i}\right)$ ^15^, ^21^, ^22^:

(2) $$\begin{array}{cc} & S\left(t|x_{i}\right)=\prod_{k=1}^{K}\;S_{k}\left(t|x_{i}\right)=\exp\left(-\int_{0}^{t}\sum_{k=1}^{K}\;h_{k}\left(u|x_{i}\right)\;\mathrm{du}\right). \end{array}$$

Each cause‐specific survival function, $S_{k}\left(t|x_{i}\right),$ can be defined with respect to the cause‐specific hazard function $h_{k}\left(t|x_{i}\right)$ as follows^23^, ^24^

(3) $$\begin{array}{cc} & S_{k}\left(t|x_{i}\right)=\exp\left(-\int_{0}^{t}h_{k}\left(u|x_{i}\right)\;\mathrm{du}\right). \end{array}$$

The integral for calculating the CIF will often not have a closed form solution and therefore may need to be obtained numerically.^23^ However, the model parameters can be exported in order to produce risk predictions in a new dataset if the model were to be independently and externally validated (see Appendix A.1.4).

The total, or all‐cause, risk of death at time t ($F_{\mathrm{all}}\left(t|x_{i}\right)$) can then be estimated as the sum of the k cause‐specific CIFs or the complement of the all‐cause survival function as shown by^16^, ^25^

(4) $$\begin{array}{cc} & F_{\mathrm{all}}\left(t|x_{i}\right)=\sum_{k=1}^{K}\;F_{k}\left(t|x_{i}\right)=1-S\left(t|x_{i}\right)\;. \end{array}$$

### 2.2 Prognostic model development

#### 2.2.1 Model format for time‐to‐event outcomes

Since we are interested in not only predicting the risk of death from colon cancer but also the risk of death from other causes, our main approach to account for competing risks is to fit a cause‐specific hazard model to each of the k=1,…,K events separately. Working in the cause‐specific setting means that the total predicted risk is constrained to not exceed 1, which is not the case for the subdistribution modelling approach.^26^, ^27^

In the applied example in Section 3, we use K = 2 and develop one model for deaths due to colon cancer (patients who die from other causes are censored) and a second model for deaths due to other causes (patients who die due to colon cancer are censored), to mirror the approach used to develop several existing risk prediction models for cancer.

The PREDICT^17^, ^18^ and QCancer^19^ prediction models use Cox proportional hazard (PH) models. These are semi‐parametric models where the baseline hazard function $h_{0k}\left(t\right)$ does not have a parametric form. However, the Breslow estimate of the baseline cumulative hazard from each model can be used to produce the cause‐specific CIFs (Section 2.1). As this will give a step function, the estimates of the baseline cumulative hazard can first be smoothed if required.^23^ The linear predictor, $\beta_{k}^{T}x_{i}$, forms the parametric component of the model and can incorporate patient characteristics ($x_{i}$) such as stage of tumour and age at diagnosis.^28^, ^29^ A Cox PH model for each cause can be written as a combination of the baseline cause‐specific hazard function and the corresponding covariate effects such that:

(5) $$\begin{array}{cc} & h_{k}\left(t|x_{i}\right)=h_{0k}\;\left(t\right)e^{\beta_{k}^{T}x_{i}}. \end{array}$$

In this notation, we assume for simplicity that the same covariates $x_{i}$ are included in each of the cause‐specific hazard models; however, this does not have to be the case.

An alternative approach is to use flexible parametric survival models (FPM). Rather than modelling on the hazard scale, these models are typically fitted on the log cumulative hazard scale and are fully parametric models.^30^ They have the following form where a restricted cubic spline function, $\zeta_{k}\left(\ln\left(t\right)|\gamma_{k},k_{0k}\right)$, with parameters $\gamma_{k}$ and knot locations $k_{0k}$, is used to model the log cumulative baseline hazard function for cause k, $\ln\left[H_{k}\left(t|x_{i}\right)\right]$ ^29^:

(6) $$\begin{array}{cc} & \ln\left[H_{k}\left(t|x_{i}\right)\right]=\zeta_{k}\left(\ln\left(t\right)|\gamma_{k},k_{0k}\right)+\beta_{k}^{T}x_{i}. \end{array}$$

FPMs are used to illustrate the approach in Section 3, however, as shown in our previous work, the methods outlined in this paper can also be applied to Cox PH models.^9^

Whichever modelling approach is taken, penalty terms could be added to the likelihood function to help address any overfitting.^31^ This would apply in the first step of temporal recalibration when the predictor effects are estimated (see Section 2.2.4). For the period analysis approach (see Section 2.2.3), including a penalty term may help with overfitting when reducing the window size as the predictor effects are estimated on this smaller subsample but we do not consider this further here.

#### 2.2.2 Standard approach (not accounting for trends in survival)

The standard approach used to develop risk prediction models does not take account of any trends in survival that occur within the development dataset. Therefore, all individuals contribute toward the estimation of both the predictor effects and the baseline hazard for each of the k models regardless of when they were diagnosed. This approach can lead to over‐estimating the risk for new patients if survival improves over this time. This is due to the higher mortality rate amongst the earliest diagnosed patients having a large influence on the overall cause‐specific hazard functions which cancels out some of the recent improvements in survival.

#### 2.2.3 Period analysis to account for trends in survival

**FIGURE 1:**
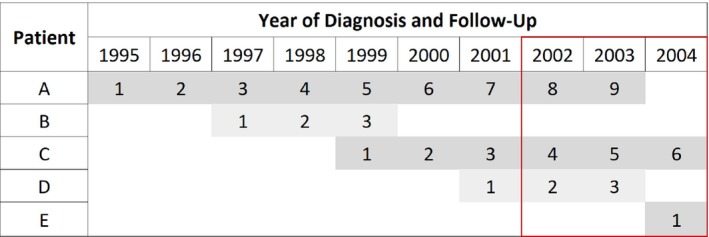
Contribution of follow‐up time from five hypothetical patients to a period analysis using a 3‐year window of 2002–2004.

Period analysis is a technique which is often used in cancer survival to produce more up‐to‐date estimates of survival by limiting the use of older data where possible.^11^, ^32^ As shown in Figure 1, a period window is defined and only the follow‐up time and events that occur during the window are included in the analysis by using delayed entry techniques.^10^, ^33^

The hazard rates at earlier time points are only estimated from patients diagnosed during or shortly before the period window (eg, Patient E) and are therefore more up‐to‐date since data from earlier diagnosed patients who experienced poorer survival are excluded (eg, Patients A and B). As patients diagnosed within the window have limited follow‐up available, estimates of the hazard rates at later time points cannot be estimated from this group alone. Therefore, delayed entry is used in order to include some information from earlier diagnosed patients. For instance, Patient A contributes toward the estimation of the hazard rates between 8 and 9 years but not at earlier time points since their follow‐up time is left truncated.

Due to maximising the use of more recent data, period analysis has been shown to produce more accurate survival estimates for new patients.^33^, ^34^, ^35^, ^36^ However, as only the events that occur during the window contribute toward the analysis, this method leads to a reduction in the sample size and number of events for model development. This may be problematic in small datasets and could lead to overfitting.^37^

The choice of the window size is a bias‐variance trade‐off where a narrower window has the potential to produce more up‐to‐date survival estimates but results in a greater reduction in the number of events and sample size as shown in Table 2. Previous studies using period analysis have used a window between 1 and 5 years.^35^, ^36^, ^38^, ^39^, ^40^, ^41^ In the applied example in Section 3, we use a window of 3 years and provide a sensitivity analysis in Appendices A.2 and A.4 using a range of window sizes to demonstrate how this choice impacts both the survival estimates and their uncertainty.

Applying period analysis when modelling on the subdistribution hazard is possible and we include an example of this in Appendix A.7. However, we would urge caution since many of the standard packages used to fit these models do not appropriately account for delayed entry.^42^ An alternative to using these packages is to first expand the data to calculate the time‐dependent weights, see Appendix A.1.3 for example Stata code.

#### 2.2.4 Temporal recalibration to account for trends in survival

Temporal recalibration is a two‐step process where the risk prediction model is first developed as usual using the full dataset to estimate the predictor effects. The model is then recalibrated by re‐estimating the baseline hazard in the period analysis window whilst constraining the predictor effects to remain the same as in the original model.^9^ This maximises the use of data as the predictor effects are estimated on the full dataset in contrast to using period analysis to develop the model where the predictor effects are estimated using data within the period window.

When developing K cause‐specific hazard models to account for competing risks, this process can be repeated on each of the models separately to ensure that the cause‐specific survival and hazard functions are as up‐to‐date as possible. The risk predictions of interest can then be obtained using the equations shown in Section 2.1.

Alternatively, if competing risks were accounted for by using the approach proposed by Fine and Gray,^20^ temporal recalibration could be applied by first fitting the standard model and then re‐estimating the baseline subdistribution hazard in the period analysis subsample whilst constraining the subdistribution hazard ratios to remain the same. Changes in the other cause mortality may affect the subdistribution hazard ratios relating to the cancer mortality (and vice versa)^43^ and hence constraining them to remain the same would be a stronger assumption than in the cause‐specific setting.

### 2.3 Assessing the calibration of risk predictions

Assessing the calibration of a prognostic model involves comparing the predicted risks to the observed risks. This section outlines approaches to estimate the observed risk, calculate calibration‐in‐the‐large and produce calibration plots.

#### 2.3.1 Nonparametric estimator of the observed risk

The Aalen–Johansen estimator, ${\hat{F}}_{k}^{\mathrm{AJ}}\left(t\right)$, is a nonparametric estimator of the cause‐specific CIF

(7) $$\begin{array}{cc} & {\hat{F}}_{k}^{\mathrm{AJ}}\left(t\right)=\sum_{j|t_{j}\leq t}\;{\hat{S}}^{\mathrm{KM}}\left(t_{j-1}\right)\frac{d_{\mathrm{kj}}}{n_{j}}, \end{array}$$

where $t_{j}$ is the $j^{\mathrm{th}}$‐ordered event time and ${\hat{S}}^{\mathrm{KM}}\left(t_{j-1}\right)$ is the all‐cause Kaplan‐Meier estimate at the previous event time.^44^, ^45^ The cause‐specific hazard rate for cause k is given by $\frac{d_{\mathrm{kj}}}{n_{j}}$, where $d_{\mathrm{kj}}$ is the number of deaths due to cause kat time $t_{j}$ and $n_{j}$ is the number of individuals at risk at time $t_{j}.$ ^15^

A nonparametric estimate of the all‐cause risk of death, ${\hat{F}}_{\mathrm{all}}^{\mathrm{KM}}\left(t\right)$, can be obtained using the all‐cause Kaplan–Meier estimate of risk ($1-{\hat{S}}^{\mathrm{KM}}\left(t\right)$).^16^, ^25^ These observed risk estimates then need to be compared with the model's predicted risks, to check if they calibrate well, as described below.

#### 2.3.2 Calibration‐in‐the‐large

In an external validation of a model, the marginal predicted cause‐specific CIFs,${\bar{F}}_{k}\left(t\right)$, can be calculated as the average predicted risk across all N individuals:

(8) $$\begin{array}{cc} & {\bar{F}}_{k}\left(t\right)=\frac{1}{N}\sum_{i=1}^{N}\;{\hat{F}}_{k}\left(t|x_{i}\right). \end{array}$$

The Aalen‐Johansen estimator can be used to quantify the observed risk and if the model is well‐calibrated, these estimates should agree closely.

The marginal predicted all‐cause CIF, ${\bar{F}}_{\mathrm{all}}\left(t\right)$, can be calculated as the sum of the K marginal cause‐specific CIFs, ${\bar{F}}_{k}\left(t\right)$:

(9) $$\begin{array}{cc} & {\bar{F}}_{\mathrm{all}}\left(t\right)=\sum_{k=1}^{K}\;{\bar{F}}_{k}\left(t\right). \end{array}$$

The calibration of the all‐cause risk predictions can then be assessed by comparing this to the all‐cause Kaplan–Meier estimate of risk.

The area under the CIFs relate to the restricted life years lost due to that cause.^46^ For example, the area under the 10‐year cause‐specific CIF for colon cancer is the restricted life years lost up to 10 years due to colon cancer. This provides an additional measure that can be used to check calibration. If the model is well‐calibrated, the area under the predicted marginal cause‐specific CIF (${\bar{F}}_{k}\left(t\right)$) should be in good agreement with the area under the Aalen‐Johansen estimator. Likewise, the area under the marginal predicted all‐cause CIF (${\bar{F}}_{\mathrm{all}}\left(t\right)$) should be similar to the area under the all‐cause Kaplan‐Meier estimate of risk.

#### 2.3.3 Calibration plots

Calibration‐in‐the‐large focuses on the overall agreement between the observed and predicted risks, but it is also important to check calibration across the entire range of predictions.^37^, ^47^ Calibration plots with calibration curves can be used to do this at each time‐point of interest. For competing risks, calibration plots can be produced for the risk of death due to each cause as well as the all‐cause (total) risk of death.

To avoid grouping individuals, pseudo‐values can be used. A pseudo‐value, ${\hat{ϴ}}_{i}$, is calculated for each individual using

(10) $$\begin{array}{cc} & {\hat{ϴ}}_{i}=n\hat{ϴ}-\left(n-1\right){\hat{ϴ}}_{-i}, \end{array}$$

where $\hat{ϴ}$ is the Aalen‐Johansen estimator when estimating cause‐specific CIFs or the all‐cause Kaplan‐Meier when estimating all‐cause survival.^48^ For the latter, $1-{\hat{ϴ}}_{i}$ gives pseudo‐values for the all‐cause risk of death. The estimator is calculated using the entire cohort ($\hat{ϴ}$) and also when excluding individual i (${\hat{ϴ}}_{-i}$) in order to calculate the pseudo‐value for individual i (${\hat{ϴ}}_{i}$).

Once the predicted risks and pseudo‐values have been calculated for each individual, it is possible to estimate a flexible calibration curve either parametrically, for example using restricted cubic splines,^49^ or non‐parametrically with a method such as the nearest neighbour smoothing.^50^ Here we use the latter by smoothing the pseudo‐values as a function of the predicted risks.

Whilst the smooth calibration plots allow calibration to be assessed visually at a particular time point of interest t, the Integrated Calibration Index (ICI) can help to quantify how well the predictions are calibrated.^49^, ^51^ The ICI is the mean absolute difference between the observed risks (obtained using the smoothed calibration curve) and the predicted risks (obtained from the prognostic model) across all individuals, where a value of zero would indicate perfect calibration. It can be calculated as follows,

(11) $$\begin{array}{cc} & {\mathrm{ICI}}_{t}=\frac{1}{N}\sum \left|{\hat{P}}_{t}^{C}-{\hat{P}}_{t}\right| \end{array}$$

where an individual's predicted risk at time t, ${\hat{P}}_{t}$, is obtained from the prognostic model and the value that the smoothed calibration curve takes at ${\hat{P}}_{t}$ gives their observed risk, ${\hat{P}}_{t}^{C}.$ ^51^ The mean of the absolute difference of ${\hat{P}}_{t}$ and ${\hat{P}}_{t}^{C}$ across all Nindividuals gives the ICI.

### 2.4 Concordance

As discussed in our original article, performing temporal recalibration on a proportional hazards model (in the non‐competing risks setting) does not affect Harrell's c‐index as the predictor effects are constrained to remain the same and therefore the ordering of the participants does not change.^9^ In the competing risks setting, Wolber's concordance index could be calculated which orders individuals based on their predicted CIF for the event of interest.^47^, ^52^ If Fine and Gray models are used with no time‐dependent effects, then the concordance index will be the same for the standard approach and temporal recalibration as the predictor effects are the same.^47^ However in the cause‐specific setting, the CIF is dependent on both cause‐specific models and therefore updating the baseline hazard of both models may result in some small changes to the ordering. Whilst we would expect the concordance index to be very similar for these methods, they will not necessarily be the same.

## 3 EXAMPLE

### 3.1 Data

We compare the three approaches (standard method – not accounting for trends in survival over time, temporal recalibration, period analysis) using an example of survival following a diagnosis of colon cancer using data from the Surveillance, Epidemiology and End Results (SEER) program which includes cancer registry data from nine registries in the United States.^53^

Model development included white and black patients diagnosed aged 18–99 with colon cancer (ICD10 codes C18.0–18.9) between 1995 and 2004 (follow‐up until December 31, 2004). Any duplicate records or patients with an unknown survival time or cause of death were excluded, as were any patients who had an incomplete date of diagnosis or death.

In our previous article, we used all available data to develop a prognostic model. However, as many prognostic models are not developed using large national databases, in this example, we restrict the analysis to a random 10% sample which leaves 4683 patients for model development. This allows us to highlight the impact of key modelling choices when working with smaller datasets—for example, this sample size allows us to demonstrate how the choice of period window to use with temporal recalibration or period analysis can affect the calibration and uncertainty of the risk predictions.

For simplicity, a complete case analysis was performed. However, these methods can be applied on multiply imputed data where the imputation models are adapted accordingly for competing risks.^54^ Rubin's rules can then be used to combine the estimates of the predictor effects and produce the risk predictions.^55^

To assess the calibration of the predictions for more recent patients, a validation dataset of all patients diagnosed in 2005 (follow‐up until December 31, 2015) was used. Table 1 presents the baseline characteristics of the development (*N* = 4683) and validation (*N* = 5504) datasets after the missing data were removed and the random 10% sample for model development was selected.

**TABLE 1:** Baseline characteristics of the development and validation datasets.

		Mean (S.D) or *N* (%)	
Variable		Development diagnosed: 1995–2004 follow‐up: until 31/12/2004	Validation diagnosed: 2005 follow‐up: until 31/12/2015
Age		70.1 (12.8)	69.3 (13.5)
Sex	Male	2283 (48.8%)	2679 (48.7%)
	Female	2400 (51.3%)	2825 (51.3%)
Ethnicity	White	4040 (86.3%)	4772 (86.7%)
	Black	643 (13.7%)	732 (13.3%)
Stage	1: Localized	1725 (36.8%)	2218 (40.3%)
	2: Regionalized	2099 (44.8%)	2261 (41.1%)
	3: Distant	859 (18.3%)	1025 (18.6%)
Grade	1: Well differentiated	542 (11.6%)	606 (11.0%)
	2: Moderately differentiated	3197 (68.3%)	3621 (65.8%)
	3: Poorly differentiated	900 (19.2%)	1170 (21.3%)
	4: Undifferentiated	44 (0.9%)	107 (1.9%)
Total		4683 (100.0%)	5504 (100.0%)

### 3.2 Methods

Three different strategies for model development were applied to both the colon cancer and other cause models: the standard approach (not accounting for trends in survival), temporal recalibration and period analysis.

Flexible parametric survival models were used to develop each of the cause‐specific hazard models using 5 degrees of freedom (6 knots) for the log cumulative baseline hazard. Using 5 degrees of freedom was thought to provide sufficient flexibility whilst not over‐parameterising the baseline. Previous simulations studies have shown flexible parametric survival models to be fairly insensitive to the number of knots used.^56^, ^57^

The colon cancer model included the following predictors and assumed proportional hazards: age (a non‐linear effect modelled with restricted cubic splines and 3 degrees of freedom) and the categorical variables of sex, ethnicity, stage and grade of tumour. All predictors except tumour grade were included in the other cause model. Stage of tumour at diagnosis would not normally be expected to impact the other cause mortality, however it was included since the effect for Stage 3 was large (hazard ratio = 2.04, see Table 3 in Appendix A.3). The magnitude of the hazard ratio was likely due to a number of patients dying due to other causes very shortly after being diagnosed with a Stage 3 tumour. These could be incidental diagnoses of cancer when patients were seriously ill in hospital and being treated for other conditions. Due to the large hazard ratio, stage at diagnosis was included in the other cause model.

Table 2 displays the sample size and number of events for developing each of the cause‐specific hazard models using the standard approach or period analysis. Although the sample size remains large even when using a 1 year period window, it includes many patients diagnosed before the window who only contribute toward the estimation of the hazard rates at later time points. Therefore, it is important to also consider the number of patients diagnosed within the window since these individuals are considered as being at risk from time zero and are the only patients to contribute toward the estimation of the hazard rates at early time points.

**TABLE 2:** Sample size and number of events for each cause when using the standard approach or period analysis for model development.

	Full sample			Diagnosed within the window		
Method	Sample size	Deaths due to colon cancer	Deaths due to other causes	Sample size	Deaths due to colon cancer	Deaths due to other causes
Standard approach	4683	1176	872	4683	1176	872
Period analysis: 5‐year window	3881	660	588	2419	440	262
Period analysis: 4‐year window	3659	531	496	1997	325	186
Period analysis: 3‐year window	3405	399	377	1505	205	113
Period analysis: 2‐year window	3167	273	266	1066	126	72
Period analysis: 1‐year window	2888	134	128	567	45	22

The use of period analysis also has a large impact on the number of events which is particularly evident in smaller datasets where only a small number of events remain in the analysis when using a narrow window. For example, when developing the models using a 3 year window, only 34% and 43% of the events were retained for the colon cancer and other cause models respectively. Although using a narrower window has the potential to produce more up‐to‐date predictions, it can also limit the number of predictor parameters that can be included due to the potential of overfitting. An advantage of temporal recalibration is that the predictor effects are estimated using the standard approach on the full dataset and only the baseline is re‐estimated using this subsample. In Section 3.4, the results using a 3 year window are presented; however, Appendix A.4 provides a sensitivity analysis when using different window sizes.

For each of the period windows, the number of events for each of the causes was similar (see Table 2), and therefore the same window width was used to develop each of the models. However, if there were a scenario where one cause of death was much more likely, for example lung cancer, where the majority of the deaths are due to the cancer, different windows could be used for each model to avoid overfitting in the model with fewer events. In the lung cancer example in Appendix A.5, a 2‐year window was used for the lung cancer model and a 4 year window was used for the other cause mortality.

To assess the calibration of the risk predictions for more recently diagnosed patients, the marginal predicted 10‐year cause‐specific and all‐cause CIFs were compared to the nonparametric equivalents in the validation dataset. The predicted and observed restricted life years lost to cancer and other causes up to 5 and 10 years were also calculated. To further assess calibration, calibration plots at 10 years were produced and the ICI for each plot was calculated.

### 3.3 Software

The analysis was performed in Stata 17 using several user‐written Stata packages: *stpm2* for fitting flexible parametric survival models,^30^ *standsurv* for producing the risk predictions,^58^ *stcompet* for calculating the Aalen‐Johansen estimates,^59^ *stpsurv* and *stpci* for calculating pseudo‐values.^48^ Example Stata code for fitting the models is provided in Appendix A.1.

### 3.4 Results

#### 3.4.1 Choice of period window

As shown in Table 2, using a period analysis approach to develop prognostic models reduces the sample size and number of events which increase the uncertainty of the predictions and may result in overfitting in some cases. Sample size criteria^60^ could be used to inform the choice of period window by calculating the minimum number of events required for a particular application and ensuring that the minimum sample size required are diagnosed within the period window.

The impact of the period window can also be informally assessed using the global shrinkage factor to determine whether there is evidence of overfitting during model development.^37^ Using the standard approach to develop the models on all available data resulted in minimal overfitting with a global shrinkage factor of at least 0.98 for each of the models.

In practice, the predictor effects from the models developed using the standard approach should be multiplied by their corresponding uniform shrinkage factor and then constrained at their shrunken values when re‐estimating the (log cumulative) baseline hazard to ensure calibration‐in‐the‐large.^37^ For temporal recalibration, the same shrunken values for the predictor effects can be used but the difference is that the baseline hazard would be re‐estimated in the period window. However, it makes little difference in this example, as the shrinkage factors are very close to 1.

In contrast, when using period analysis, the predictor effects are estimated on the subsample of data defined by the window. Whilst the global shrinkage factor was at least 0.97 when using a 3 year window, using increasingly narrow windows led to a greater degree of overfitting, see Table 3. For simplicity here, no adjustment for overfitting was made since the shrinkage factors for all the models included in the main analysis are close to 1 but in principle the predictor effects should be constrained at the shrunken values and the baseline re‐estimated. This is most important in small sample sizes where the shrinkage factor becomes far from 1.

**TABLE 3:** Global shrinkage factor for each of the cause‐specific models.

Method	Colon cancer	Other causes
Standard approach (or temporal recalibration)	0.99	0.98
Period analysis: 5‐year window	0.99	0.98
Period analysis: 4‐year window	0.99	0.98
Period analysis: 3‐year window	0.98	0.97
Period analysis: 2‐year window	0.97	0.95
Period analysis: 1‐year window	0.93	0.90

In addition, the standard error of the predictor effects can also be examined. For example, the standard errors of the log hazard ratios are approximately twice as large when using a period analysis window of 3 years in comparison to the standard approach, see Tables 2 and 3 in Appendix A.3.

**FIGURE 2:**
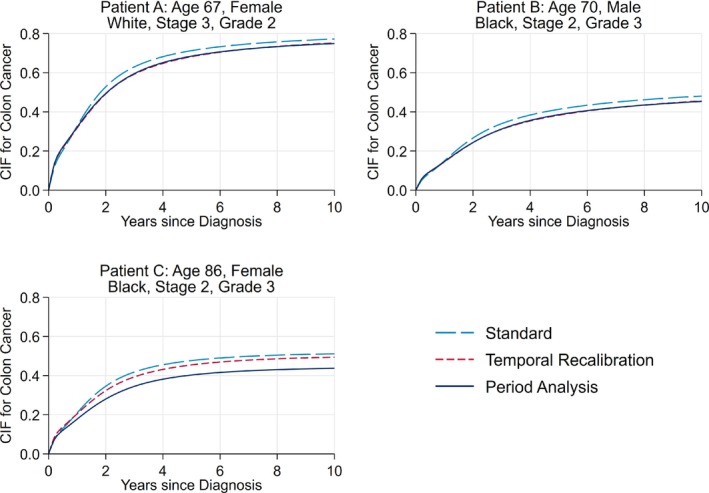
Comparison of the predicted risk of death due to colon cancer of three patients with different covariate patterns. The predictions for temporal recalibration and period analysis are from the models which used a 3‐year window. The predictions from temporal recalibration and period analysis overlay almost exactly for Patients A and B.

In addition to minimising overfitting, it is also important to consider the uncertainty in estimating the baseline of the model.^60^ One approach to assess this could be to fit a model without any predictors and estimate the cause‐specific survival functions with a 95% confidence interval to gain an understanding of the impact that using a smaller window may have due to there being fewer events. Given that temporal recalibration only estimates the baseline and keeps the covariates fixed it gives an informal guide to the impact of the choice of window size. Figures 1 and 2 in Appendix A.2 show the impact of changing the window on the width of these intervals, and hence the uncertainty of these estimates.

The choice of period window could then be guided based on a combination of the global shrinkage factor, the uncertainty in the predictor effects and the uncertainty in the baseline. In temporal recalibration, only the baseline is estimated in the period analysis subsample and therefore the most important consideration would be the precision for estimating the baseline since the predictor effects are estimated on the full dataset.

#### 3.4.2 Calibration

Accounting for trends in survival during model development can lead to updated risk predictions for individuals. Figure 2 displays the predicted risk of death due to colon cancer for three patients with varying characteristics. As can be seen, using temporal recalibration or period analysis produced predictions that were around 3 percentage points lower than using the standard approach for Patients A and B. For many covariate patterns, the risk predictions from temporal recalibration and period analysis were similar; however, for Patient C and other more elderly patients, using period analysis produced the lowest risk predictions that were around 7 percentage points lower than the standard method.

**FIGURE 3:**
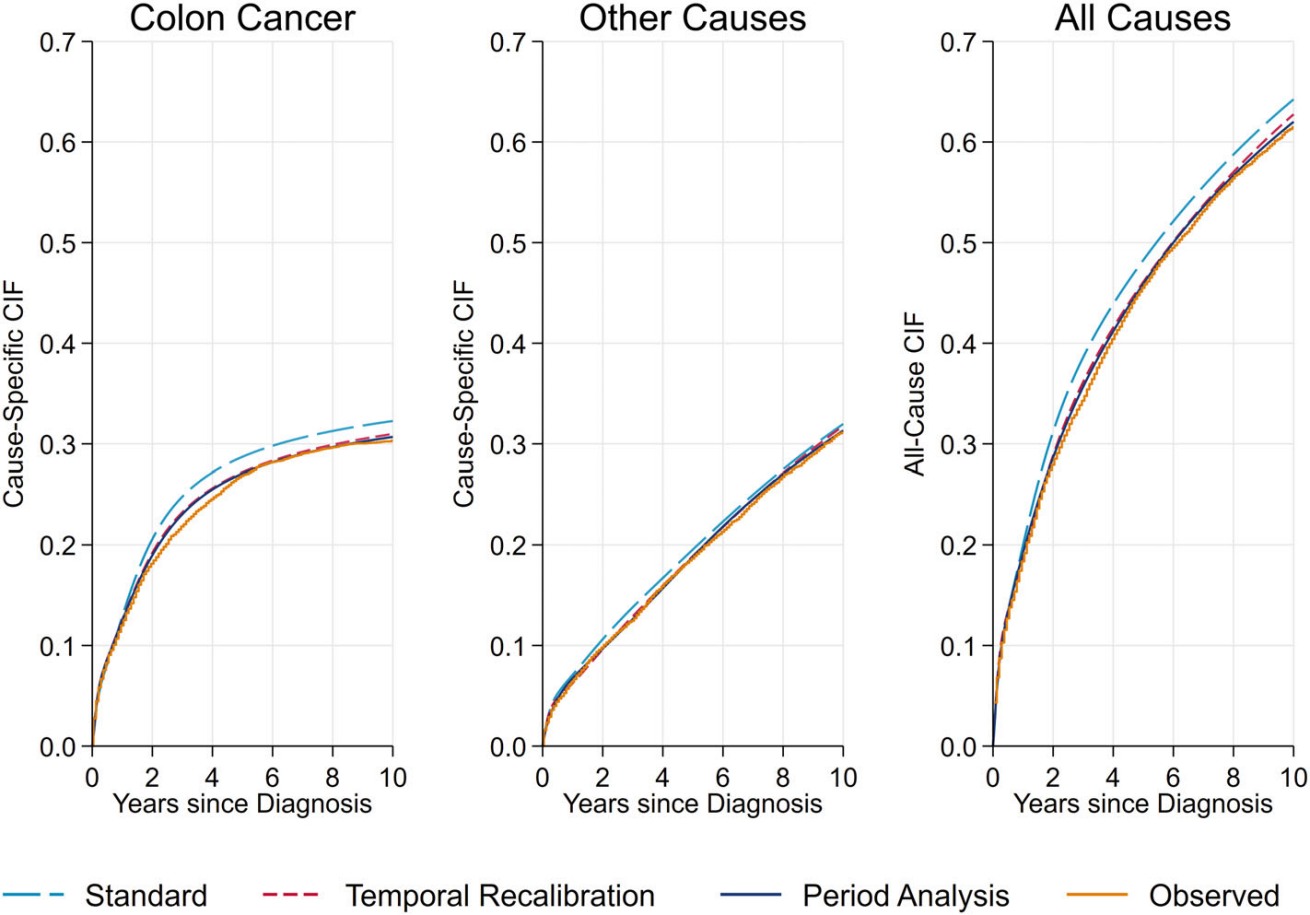
Comparison of the predicted and observed marginal cause‐specific CIFs. The predictions for temporal recalibration and period analysis overlay almost exactly and are from the models which used a 3‐year window.

Figure 3 compares the predicted marginal (average) CIFs for the validation dataset (patients diagnosed in 2005) to the observed nonparametric estimates. Using the standard approach to develop each model led to an over‐estimation of 0.019, 0.008 and 0.028 for the 10‐year marginal CIFs for colon cancer, other causes and all causes respectively, see Table 4. Using temporal recalibration or period analysis improved the calibration of the predicted risks across the full range of follow‐up, where particular improvements can be seen in the predicted risk of death due to colon cancer and the total risk of death. Very similar results were found when modelling on the subdistribution hazard scale as shown in Appendix A.7.

**TABLE 4:** Difference between the predicted and observed CIFs at 5 and 10 years post diagnosis with a 95% confidence interval.

Time Point	Method	Colon Cancer	Other Causes	All Causes
5 years	Standard approach	0.019 [0.007, 0.030]	0.009 [−0.001, 0.019]	0.028 [0.014, 0.041]
	Temporal recalibration	0.003 [−0.008, 0.015]	0.003 [−0.008, 0.013]	0.006 [−0.007, 0.019]
	Period analysis	0.002 [−0.010, 0.014]	0.002 [−0.009, 0.012]	0.004 [−0.009, 0.017]
10 years	Standard approach	0.019 [0.007, 0.032]	0.008 [−0.004, 0.020]	0.028 [0.015, 0.041]
	Temporal recalibration	0.007 [−0.006, 0.019]	0.006 [−0.006, 0.018]	0.013 [0.000, 0.026]
	Period analysis	0.004 [−0.009, 0.016]	0.002 [−0.011, 0.014]	0.005 [−0.008, 0.018]

*Note*: The predictions from using a 3 year window are presented for temporal recalibration and period analysis.

The predictions at 5 years are slightly better calibrated than at 10 years for the models developed using temporal recalibration or period analysis. The predictions at earlier time points are likely to always be better calibrated since it is possible to estimate them using more recent data. For example, the 10 year hazard rates can only be estimated from patients diagnosed in 1995 since these are the only individuals who have sufficient follow‐up time. In contrast, the 5 year hazard rates are estimated from more recently diagnosed patients (diagnosed between 1997 and 2000) in the temporal recalibration and period approach and the estimates are therefore more up‐to‐date.

**FIGURE 4:**
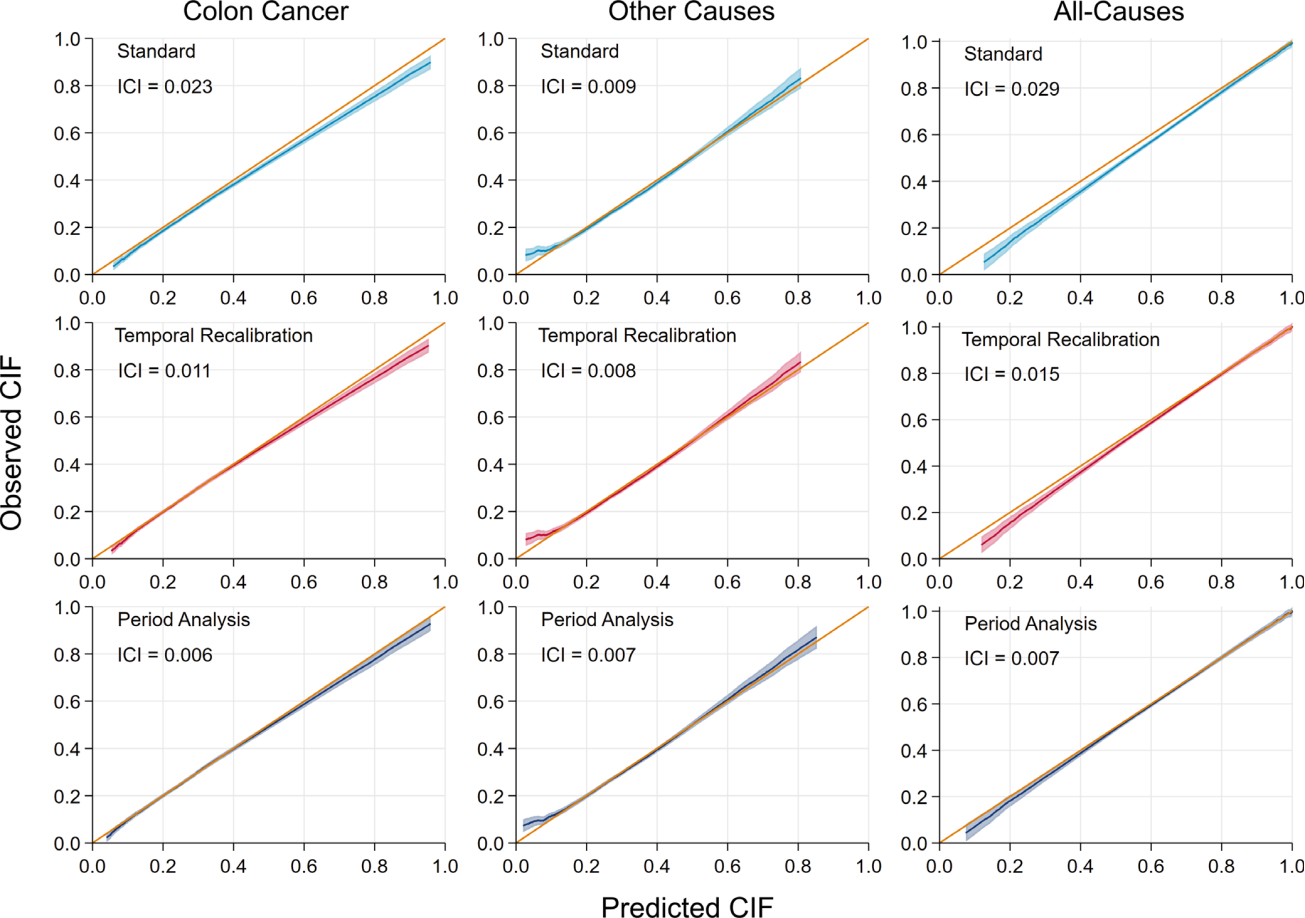
Calibration plots at 10 years for each of the cause‐specific CIFs and the total risk. The results from using a 3‐year window are presented for the temporal recalibration and period analysis models.

Improvements in calibration can also be seen in the calibration plots in Figure 4, where using either temporal recalibration or period analysis led to a reduction in the Integrated Calibration Index. For all methods, the risk predictions for death due to other causes under‐estimated the risk in the low‐risk patients. However, this only affected a small number of patients as only 49 had a cause‐specific CIF less than 0.05.

**FIGURE 5:**
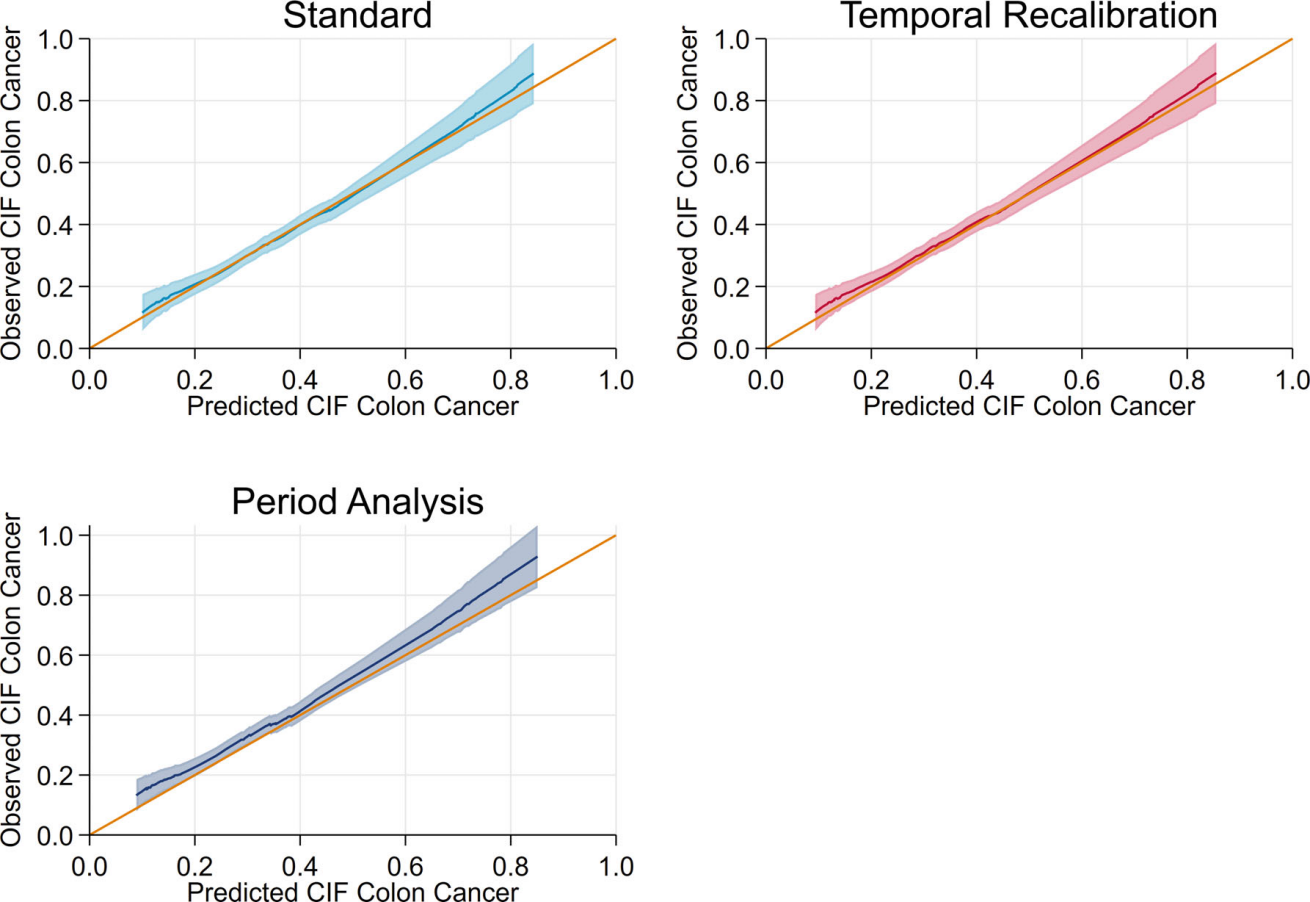
Calibration plots at 10 years for the cause‐specific CIF due to colon cancer for patients aged 85 and over at diagnosis. The results from using a 3‐year window are presented for the temporal recalibration and period analysis models.

The marginal CIFs using different window sizes for temporal recalibration and period analysis are presented in Figures 3–6 in Appendix A.4. Whilst using a 5 year window led to greater precision, a 2 year window further improved the calibration of the predictions. This demonstrates the bias‐variance trade‐off where better calibrated risk predictions can be produced using a narrower window at the cost of greater uncertainty. When selecting the window, it is important to ensure that there are a sufficient number of events to estimate the baseline (and the predictor effects if using period analysis) reliably.

The area under the CIFs in Figure 3 relates to the restricted life years lost up to 10 years.^46^ By improving the calibration of the risk predictions, the estimates of the life years lost due to colon cancer and the total life years lost agreed more closely with the observed nonparametric estimators as shown in Table 5. It is important to account for trends in survival in each of the cause‐specific models. If only one of the models were temporally recalibrated, the other model would remain miscalibrated and although the calibration of the total risk predictions would improve, the proportion of life years lost to each cause would be incorrect.

**TABLE 5:** Restricted life years lost up to 5 and 10 years and the restricted life years lost due to colon cancer for the validation dataset.

Time Point	Method	Restricted life years lost	Restricted life years lost due to cancer (%)
5 years	Aalen‐Johansen	1.47	0.92 (62.6%)
	Standard approach	1.61	1.02 (63.2%)
	Temporal recalibration	1.52	0.97 (63.6%)
	Period analysis	1.51	0.96 (63.5%)
10 years	Aalen‐Johansen	4.21	2.39 (56.7%)
	Standard approach	4.46	2.56 (57.4%)
	Temporal recalibration	4.27	2.44 (57.0%)
	Period analysis	4.25	2.42 (57.0%)

*Note*: The results for temporal recalibration and period analysis were from using a 3‐year window.

Although the performance of temporal recalibration and period analysis was similar, differences in risk predictions were identified for certain covariate patterns, in particular for the most elderly patients where period analysis produced much lower risk predictions for death due to colon cancer (eg, Patient C, Figure 2). This was further investigated by producing calibration plots that only included those aged 85 and over at diagnosis, see Figure 5. Here, it can be seen that using period analysis under‐estimated the CIF whilst using temporal recalibration produced better calibrated risk predictions. These differences may be due to the greater uncertainty at which the hazard ratios for age can be estimated in period analysis, particularly when there is sparser data in the upper tail of the age distribution. For example, there were 532 patients aged 85 and over in the model development dataset. However, this reduced to 315 when using period analysis with a 3 year window since many of these older patients have short survival times and therefore did not survive into the window to be included in the analysis.

## 4 DISCUSSION

Not accounting for trends in survival over calendar time when developing prognostic models can lead to miscalibrated risk predictions for new patients. In this example, not accounting for the improvements in survival following a diagnosis of cancer resulted in over‐estimating the risk and consequently under‐estimating the long‐term survival when the model was temporally validated in the same population but with more recently diagnosed patients. Using period analysis can lead to better calibrated predictions but results in a reduction in the sample size and number of events that can be used to develop the model.

Temporal recalibration can also be used to produce more up‐to‐date risk predictions by first estimating the predictor effects on the full dataset and then re‐estimating the baseline hazard using delayed entry techniques to capture improvements in survival. Due to the lack of long‐term follow‐up data for the most recently diagnosed patients, standard recalibration techniques can often only be applied once new data are available. For example, the most recently diagnosed patients in the development dataset may have a maximum of 3 years of follow‐up and therefore it would not be possible to update the 10‐year risk estimates without additional follow‐up information. In contrast, temporal recalibration simply uses a subset of the original data to update the model parameters. This allows improvements in survival to be accounted for at the model development stage. This method can be extended into a competing risk setting by repeating this process for each of the cause‐specific hazard models separately and then using these more up‐to‐date hazard and survival functions to produce the risk predictions.

In the applied example for colon cancer survival, we showed that using either period analysis or temporal recalibration improved the calibration‐in‐the‐large and the calibration within different risk groups both for the risk due to colon cancer and the total risk of death. In this case, the development data only spanned 10 years, however, if older data were also included, or there were larger improvements in survival over this time period, using delayed entry methods would have a larger impact.

In datasets with a large sample size and number of events, period analysis is likely to perform very well. In addition to providing an updated baseline, more up‐to‐date predictor effects can be obtained if there have been substantial changes over time, since they are estimated on a recent subsample of data. However, overfitting will be more problematic with period analysis in situations with small sample sizes. In this particular example, although there were only 399 and 377 events in each cause‐specific hazard model when a 3‐year period window was used, the prognostic model was relatively simplistic and only included a small number of predictor parameters with no interaction terms or time‐dependent effects. Therefore, the amount of shrinkage remained small despite the number of events being reduced by around 60% when using period analysis. If a more complex model were to be developed using this dataset, the issue of overfitting would be more problematic when using period analysis in comparison to estimating the predictor effects on the full dataset.

Overfitting would also be an issue when using period analysis in small datasets and in these settings, shrinkage and penalisation methods may not always be reliable.^61^ Developing prognostic models in competing risk settings can be particularly complex since a sufficient number of events must be available to develop each of the cause‐specific hazard models in order to produce well‐calibrated risk predictions. Therefore, even if the event of interest is common, the competing events may occur less frequently and hence overfitting may be present in at least one of the models. An example of this could be lung cancer, where there were approximately four times as many deaths due to cancer than other causes (see Appendix A.5).

In contrast, temporal recalibration utilises all available patient data to estimate the predictor effects which will likely make this method more stable in these settings. As the predictor effects can be estimated more precisely, this method is particularly useful when there are rare covariate patterns. For example, temporal recalibration produced better calibrated risk predictions for patients aged over 85 at diagnosis. Therefore, whilst period analysis will likely perform very well in large datasets where overfitting is not an issue, temporal recalibration is a more appropriate method in smaller datasets. With either method, careful thought is required when selecting an appropriate period window to ensure that there are a sufficient number of events to estimate the model parameters reliably.

In our main analysis we focused on modelling in the cause‐specific setting but we also demonstrate in Appendix A.7 that very similar improvements in calibration can be made when using temporal recalibration or period analysis with Fine and Gray models. Although it is possible to apply these approaches in this setting, caution must be taken as many of the standard software packages used to fit models on the subdistribution hazard scale do not appropriately account for delayed entry.^42^

In Section 3, we only showed a simple example using FPMs to illustrate the process of fitting these types of models under a complete case analysis and assuming proportional hazards. However, these methods can be used in a range of model formats including Cox PH models, in conjunction with multiple imputation and when the PH assumption is not valid.

Using temporal recalibration in a competing risks setting is directly applicable to existing prognostic models for cancer such as PREDICT Breast,^17^ PREDICT Prostate^18^ and QCancer Colorectal Survival,^19^ all of which use one cause‐specific hazard model for deaths due to the cancer of interest and a second for deaths due to other causes. Using temporal recalibration could provide a suitable approach to update these types of models without the need for any additional data. However, when models are developed using databases that are regularly updated, incorporating this more recent data when performing temporal recalibration has the potential to make additional improvements in calibration.

In addition, temporal recalibration could also be used if models are to be continually updated over time when new data become available.^62^ For example, in our previous article, we demonstrated that if the predictor effects are stable over time, the baseline can simply be updated in a more recent period window in the extended dataset that includes both the original and new data.^9^ We also showed an alternative approach where the model was first re‐fitted with, for example, the latest 10 years of data (using the standard approach). This allows new estimates of the predictor effects to be calculated which is advantageous if there have been any changes over time. This model can then be temporally recalibrated to update the baseline. Both of these methods extend naturally into the competing risk settings by updating each cause‐specific model separately. In summary, temporal recalibration can be applied in a wide range of settings and is well‐suited for developing risk prediction models with competing risks, particularly when cause‐specific hazard models are used. By accounting for improvements in survival at the model development stage, better calibrated risk predictions for new patients can be produced.

**Recommendations**

- In small datasets, temporal recalibration is our preferred approach since only the baseline is estimated using the recent subsample of data. As the predictor effects are estimated using all the data this limits overfitting in comparison to period analysis.
- In large datasets, both temporal recalibration and period analysis are likely to perform well in estimating a more up‐to‐date baseline hazard. Due to the size of the data there will be a large number of events even when using period analysis and so overfitting should be minimal using either method. In settings where the predictor effects also change substantially over time, using period analysis would be advantageous since the predictor effects are estimated using the more recent subsample and will therefore be more up‐to‐date.

## CONFLICT OF INTEREST STATEMENT

Sarah Booth, Lucinda Archer, Joie Ensor, Richard D. Riley, Paul C. Lambert, Mark J. Rutherford: None. Sarwar I. Mozumder: Employed by Roche Products Ltd and AstraZeneca for work unrelated to this research during the drafting of the manuscript.

## FUNDING INFORMATION

Sarah Booth was supported by Cancer Research UK project grant (C14183/A29739). This work was supported by Health Data Research UK, an initiative funded by UK Research and Innovation, Department of Health and Social Care (England) and the devolved administrations, and leading medical research charities. Sarwar I. Mozumder was supported by the National Institute for Health Research (NIHR Advanced Fellowship, Dr Sarwar Mozumder, NIHR300100). Paul C. Lambert received support from the Swedish Cancer Society (Cancerfonden) (grant number 2018/744) and the Swedish Research Council (Vetenskapsrådet) (grant number 2017‐01591). Mark J. Rutherford received support from a Cancer Research UK project grant (C41379/A27583).

## Supporting information


**Data S1.** Supporting Information

## Data Availability

The data that support the findings of this study are available through the Surveillance, Epidemiology, and End Results Program (SEER). Access to the database can be requested here: https://seer.cancer.gov/data/access.html.
