# Supplementary material for: Using temporal recalibration to improve the calibration of risk prediction models in competing risk settings when there are trends in survival over time
Source: Stat Med. 2023 Sep 13;42(27):5007–24. doi: 10.1002/sim.9898 (PMC10946485; doi:10.1002/sim.9898)
Supplement: Supplementary file 1 — Data S1. Supporting Information [file SIM-42-5007-s001.pdf]

Appendix

A.1. Stata code

A.1.1 Temporal recalibration program

```
program define recalibrate, rclass
	syntax [varlist(default=none)], [model(string)]
	if "`model'"!="" {
		estimates restore `model'
	}

	* Main Effects
	local savedmodel=`"`e(cmdline)'"'
	gettoken modelcov options: savedmodel, parse(,)
	gettoken model cov: modelcov
	fvexpand `cov'

	foreach var in `r(varlist)' {
		local c = _b["`var'"]
		constraint free
		constraint `r(free)' _b["`var'"] = `c'
		local constraint_list "`constraint_list' `r(free)'"
	}

	* Time Dependent Effects (if any) - for flexible parametric survival models
	if "`e(cmd)'"=="stpm2" {
		foreach var in `e(tvc)' {
			forvalues i=1/`e(df_`var')' {
				local c = _b[_rcs_`var'`i']
				constraint free
				constraint `r(free)' _b[_rcs_`var'`i'] = `c'
				local constraint_list "`constraint_list' `r(free)'"
			}
		}
	}

	* Return Constraint List
	return local constraints = "`constraint_list'"

	* Knot Locations - for flexible parametric survival models
	if "`e(cmd)'"=="stpm2" {
		return local knots = e(bhknots)
		return local bknots = e(boundary_knots)
		foreach var in `e(tvc)' {
			return local tvcknots_`var' = e(tvcknots_`var')
		}
	}
end
```

A.1.2 Temporal recalibration in the cause-specific setting

Step 1: Estimate the predictor effects using the standard approach

```
stset exit, origin(dx) fail(cod==1) scale(365.24) exit(time mdy(12,31,2004))

stpm2 agercs* female black stage2 stage3 grade2 grade3 grade4, ///
scale(hazard) df(5)noorthog
estimates store cancer

stset exit, origin(dx) fail(cod==2) ///
scale(365.24) exit(time mdy(12,31,2004))
```

```
stpm2 agercs* female black stage2 stage3, scale(hazard) df(5) noorthog
estimates store other
```

Step 2: Recalibrate the baseline

```
stset exit, origin(dx) fail(cod==1) scale(365.24) ///
enter(time mdy(1,1,2002)) exit(time mdy(12,31,2004))

recalibrate, model(cancer)
stpm2 agercs* female black stage2 stage3 grade2 grade3 grade4, scale(hazard) ///
noorthog bknots("`r(bknots)'") knots("`r(knots)'") constraints("`r(constraints)'")
estimates store temp_recal_cancer

stset exit, origin(dx) fail(cod==2) scale(365.24) ///
enter(time mdy(1,1,2002)) exit(time mdy(12,31,2004))

recalibrate, model(other)
stpm2 agercs* female black stage2 stage3, scale(hazard) ///
noorthog bknots("`r(bknots)'") knots("`r(knots)'") constraints("`r(constraints)'")
estimates store temp_recal_other
```

Step 3: Estimation of the marginal cause-specific cumulative incidence functions

```
range timevar10 0 10 1001

standsurv if val==1, crmodels(temp_recal_cancer temp_recal_other) cif ///
timevar(timevar10) atvar(F) verbose at1(.)
```

Step 4: Estimation of each individual's cause-specific cumulative incidence function at 10 years

```
gen t10 = 10 in 1

standsurv if val==1, genind(F10) crmodels(temp_recal_cancer temp_recal_other) ///
cif timevar(t10) verbose at1(.)
```

A.1.3 Temporal recalibration in the subdistribution hazard setting

As not all of the standard packages to fit Fine and Gray models account for delayed entry appropriately, these models can alternatively be fitted by first expanding the data to create the time-dependent weights and then fitting a standard flexible parametric survival model or Cox model.

Step 1: Estimate the predictor effects using the standard approach

The number of events in the cause of death variable must match the output in *stset* in order for *stcrprep* to run. Since this will not be the case when *exit()* is specified in *stset,* new variables can be created to store the exact survival times and the event indicator.

```
rename cod cod_temp

stset exit, origin(dx) fail(cod_temp=1,2) id(id) scale(365.24) ///
exit(time min(dx+10*365.24,mdy(12,31,2004)))

keep if _st==1
gen surv = _t
gen cod = 0
replace cod = cod_temp if _d==1

stset surv, fail(cod=1,2) id(id)
stcrprep, events(cod) keep(agercs* female black stage2 stage3 grade2 grade3 grade4)
gen event = failcode == cod
stset tstop [iw=weight_c], failure(event==1) enter(tstart)

stpm2 agercs* female black stage2 stage3 grade2 grade3 grade4 if failcode==1, ///
scale(hazard) df(5) noorthog
estimates store cancer
```

```
stpm2 agercs* female black stage2 stage3 grade2 grade3 grade4 if failcode==2, ///
scale(hazard) df(5) noorthog
estimates store other
```

Step 2: Recalibrate the baseline

The original dataset then needs to be reloaded again so that new time-dependent weights can be calculated for use in the recalibration step.

```
rename cod cod_temp

stset exit, origin(dx) fail(cod_temp==1,2) scale(365.24) ///
enter(time mdy(1,1,2002)) exit(time min(dx+10*365.24,mdy(12,31,2004)))

keep if _st==1
gen surv = _t
gen t0 = _t0
gen cod = 0
replace cod = cod_temp if _d==1

stset surv, enter(t0) fail(cod=1,2) id(id)
stcrprep, events(cod) ///
keep(agercs* female black stage2 stage3 grade2 grade3 grade4)
gen event = failcode == cod
```

When there is delayed entry this need to be incorporated in the final weights by multiplying *weight_c* by *weight_t* (when there is no left truncation, weight_t = 1).

```
gen weight_combined = weight_c*weight_t
stset tstop [iw=weight_combined], failure(event==1) enter(tstart)

constraints_all_models, model(cancer)
stpm2 agercs* female black stage2 stage3 grade2 grade3 grade4 ///
if failcode==1, scale(hazard) noorthog bknots("`r(bknots)'") ///
knots("`r(knots)'") constraints("`r(constraints)'")
estimates store temp_recal_cancer

constraints_all_models, model(other)
stpm2 agercs* female black stage2 stage3 grade2 grade3 grade4 ///
 if failcode==2, scale(hazard) noorthog bknots("`r(bknots)'") ///
knots("`r(knots)'") constraints("`r(constraints)'")
estimates store temp_recal_other
```

Step 3: Estimation of the marginal cause-specific cumulative incidence functions

The original dataset then needs to be reloaded again in order to make the predictions.

```
gen _t0=.
gen _t=.
gen _d=.
gen weight_c=.
gen weight_combined=.
range timevar10 0 10 1001

standsurv if val==1, failure timevar(timevar10) atvar(F_temp_recal_cancer) at1(.)
standsurv if val==1, failure timevar(timevar10) atvar(F_temp_recal_other) at1(.)
```

Step 4: Estimation of each individual's cause-specific cumulative incidence function at 10 years

```
gen t10 = 10

estimates restore temp_recal_cancer
predict F10_temp_recal_cancer if val==1, failure timevar(t10)
estimates restore temp_recal_other
predict F10_temp_recal_other if val==1, failure timevar(t10)
```

A.1.4 Producing risk predictions from a previously published FPM (cause-specific)

For k = 2, the equations for calculating the CIFs can be written as follows:

$$F_1(t|\boldsymbol{x}_i) = \int_0^t S_1(t|\boldsymbol{x}_i)\, S_2(t|\boldsymbol{x}_i)\, h_1(u|\boldsymbol{x}_i)\, du$$

$$F_2(t|\boldsymbol{x}_i) = \int_0^t S_1(t|\boldsymbol{x}_i)\, S_2(t|\boldsymbol{x}_i)\, h_2(u|\boldsymbol{x}_i)\, du$$

A flexible parametric survival model with 5 degrees of freedom has the following form:

$$\ln[H_k(t|\boldsymbol{x}_i)] = \gamma_{k0} + \gamma_{k1}z_{k1} + \gamma_{k2}z_{k2} + \gamma_{k3}z_{k3} + \gamma_{k4}z_{k4} + \gamma_{k5}z_{k5} + \boldsymbol{\beta}_{\boldsymbol{k}}^{\boldsymbol{T}}\boldsymbol{x}_{ki}$$

The model coefficients can be used to calculate the cause-specific survival and hazard functions required in the estimation of the CIFs.

$$S_k(t|\boldsymbol{x}_i) = \exp\left[-\exp\left[\gamma_{k0} + \gamma_{k1}z_{k1} + \gamma_{k2}z_{k2} + \gamma_{k3}z_{k3} + \gamma_{k4}z_{k4} + \gamma_{k5}z_{k5} + \boldsymbol{\beta}_{\boldsymbol{k}}^{\boldsymbol{T}}\boldsymbol{x}_{ki}\right]\right]$$

$$h_k(t|\boldsymbol{x}_i) = \frac{d}{dt}[H_k(t|\boldsymbol{x}_i)]$$

The code below can be used when the baseline splines are not orthogonalised (when the `noorthog` option has been used) but it can be extended for use with models with orthogonalised baseline splines.

Cancer model (age modelled with restricted cubic splines centred on age 75, sex, ethnicity, stage of tumour at diagnosis and grade of tumour at diagnosis):

```
stpm2 agercs* female black stage2 stage3 grade2 grade3 grade4, scale(hazard) ///
df(5) noorthog
```

Other cause model (age modelled with restricted cubic splines centred on age 75, sex, ethnicity and stage of tumour at diagnosis):

```
stpm2 agercs* female black stage2 stage3, scale(hazard) df(5) noorthog
```

The following code shows how to produce the cause-specific CIF for cancer and for other causes for a white woman aged 75 at diagnosis, with a Stage 1 and Grade 2 tumour. This requires the knot locations (on the log scale), the coefficients of the parameters used to model the baseline and the log hazard ratios for sex and Grade 2 (the values of all other predictors are zero).

	Cancer	Other causes
Knot locations (log scale)	-3.178026451465276<br>-1.568614206711717<br>-0.3448395300313559<br>0.3184538866824854<br>0.8649977977399275<br>2.164280474836114	-3.178026451465276<br>-1.232143803521468<br>0.117781894853338<br>0.9328200618778831<br>1.513293761447519<br>2.27298446164487
γ_0 (_b[cons])	-2.7089619	-1.5794709
γ_1 (_b[_rcs1])	1.2420875	1.1070539
γ_2 (_b[_rcs2])	0.22608408	0.1629823
γ_3 (_b[_rcs3])	-0.36047962	-0.20055125
γ_4 (_b[_rcs4])	0.04613936	0.18525938
γ_5 (_b[_rcs5])	0.22518882	-0.21348543
Log hazard ratio: Female	-0.15246199	-0.18401639
Log hazard ratio: Grade 2	0.25815843	N/A

```
range time10 0 10 1001
gen double lnt = ln(time10)

rcsgen lnt, knots(-3.178026451465276 -1.568614206711717 -0.3448395300313559
0.3184538866824854 0.8649977977399275 2.164280474836114) gen(z) dgen(dz)

gen double h1 = exp(-2.7089619 + 1.2420875*z1 + 0.22608408*z2 ///
-0.36047962*z3 + 0.04613936*z4 + 0.22518882*z5 ///
-0.15246199 + 0.25815843)*(1.2420875*dz1 + 0.22608408*dz2 ///
-0.36047962*dz3 + 0.04613936*dz4 + 0.22518882*dz5)*1/time10

gen double s1 = exp(-exp(-2.7089619 + 1.2420875*z1 + 0.22608408*z2 ///
-0.36047962*z3 + 0.04613936*z4 + 0.22518882*z5 -0.15246199 + 0.25815843))
replace s1 = 1 in 1

rcsgen lnt, knots(-3.178026451465276 -1.232143803521468 0.117781894853338
0.9328200618778831 1.513293761447519 2.27298446164487) gen(y) dgen(dy)

gen double h2 = exp(-1.5794709 + 1.1070539*y1 + 0.1629823*y2 ///
-0.20055125*y3 + 0.18525938*y4 -0.21348543*y5 ///
-0.18401639)*(1.1070539*dy1 + 0.1629823*dy2 -0.20055125*dy3 + 0.18525938*dy4 ///
-0.21348543*dy5)*1/time10

gen double s2 = exp(-exp(-1.5794709 + 1.1070539*y1 + 0.1629823*y2 ///
-0.20055125*y3 + 0.18525938*y4 -0.21348543*y5 -0.18401639))
replace s2 = 1 in 1

gen double f1 = s1*s2*h1
replace f1 = 0 in 1
integ f1 time10, gen(CIF_cancer)

gen double f2 = s1*s2*h2
replace f2 = 0 in 1
integ f2 time10, gen(CIF_other)
```

References

Lambert, P. C. & Royston, P. Further Development of Flexible Parametric Models for Survival Analysis, The Stata Journal, 9(2):265-290, 2009

Lambert, Paul C. The estimation and modelling of cause-specific cumulative incidence functions using time-dependent weights, The Stata journal, 17:181-207, 2017

A.2. Sample size considerations

One approach to investigate the increase in uncertainty when using period analysis is to fit the cause-specific hazard models without any predictors (null model) and to estimate the cause-specific survival with a 95% confidence interval. The bias-variance trade off can be seen in Table 1 as using a narrower window increases the width of the confidence intervals whilst providing higher, and potentially more up-to-date, 10-year cause-specific survival estimates.

Table 1: Estimate of the cause-specific survival function and 95% confidence interval (CI) at 10 years after diagnosis and width of the CI when using the standard method or using period analysis

**Model**	**Colon Cancer**		**Other Causes**	
	$\hat{S}_k(10)$	**Width CI**	$\hat{S}_k(10)$	**Width CI**
Standard	0.624 [0.603, 0.646]	0.043	0.556 [0.524, 0.590]	0.067
5 year window	0.645 [0.623, 0.672]	0.049	0.565 [0.531, 0.602]	0.070
4 year window	0.655 [0.630, 0.682]	0.052	0.566 [0.531, 0.603]	0.072
3 year window	0.658 [0.629, 0.699]	0.059	0.576 [0.539, 0.576]	0.077
2 year window	0.661 [0.626, 0.697]	0.070	0.571 [0.528, 0.617]	0.088
1 year window	0.673 [0.626, 0.723]	0.098	0.594 [0.538, 0.656]	0.118

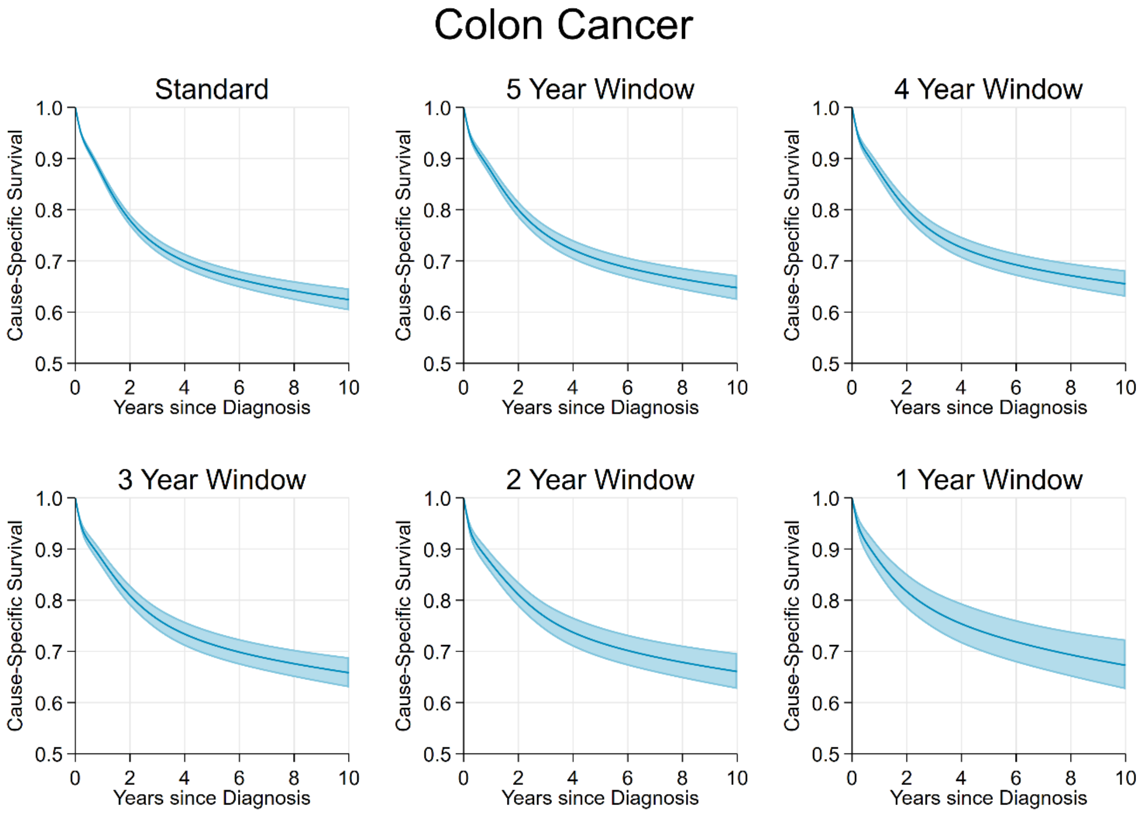

Figure 1: Comparison of the cause-specific survival function and 95% confidence interval from the colon cancer model when developing it using the standard method or period analysis with different window widths

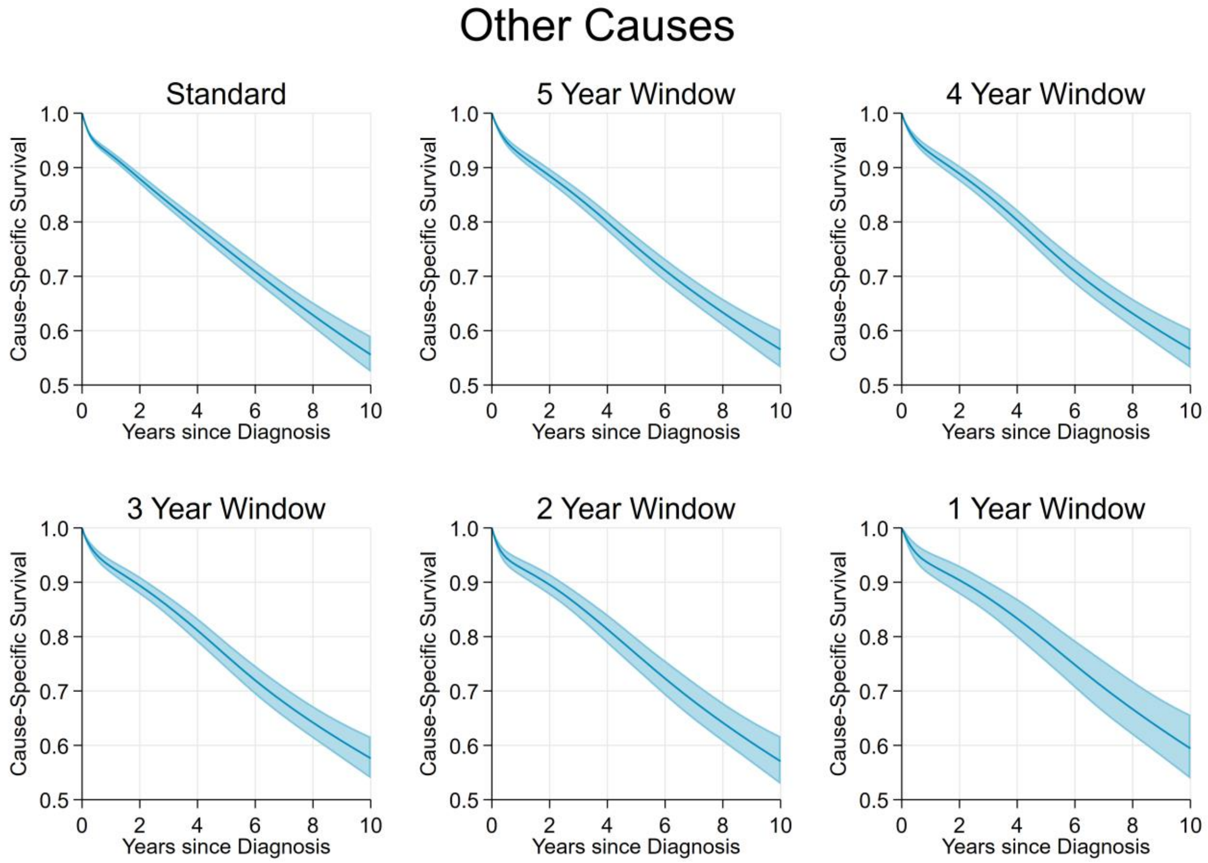

Figure 2: Comparison of the cause-specific survival function and 95% confidence interval for the other cause model when developing it using the standard method or period analysis with different window widths

A.3. Table of coefficients from each of the cause-specific hazard models

Table 2: Log hazard ratios (standard error) for the cause-specific hazard model for colon cancer

Variable		Standard	Period
Age	Splines term 1	0.299 (0.031)	0.333 (0.054)
	Splines term 2	-0.120 (0.030)	-0.071 (0.053)
	Splines term 3	-0.034 (0.029)	-0.019 (0.052)
Sex	Male	–	–
	Female	-0.152 (0.059)	-0.264 (0.102)
Ethnicity	White	–	–
	Black	0.346 (0.078)	0.326 (0.134)
Stage	1: Localised	–	–
	2: Regionalised	1.051 (0.100)	1.171 (0.164)
	3: Distant	2.964 (0.100)	3.050 (0.168)
Grade	1: Well differentiated	–	–
	2: Moderately differentiated	0.258 (0.129)	0.208 (0.208)
	3: Poorly differentiated	0.713 (0.136)	0.520 (0.223)
	4: Undifferentiated	0.840 (0.244)	0.844 (0.457)

Table 3: Log hazard ratios (standard error) for the cause-specific hazard model for other causes

Variable		Standard	Period
Age	Splines term 1	0.834 (0.058)	0.903 (0.094)
	Splines term 2	-0.110 (0.058)	-0.059 (0.094)
	Splines term 3	0.038 (0.039)	-0.004 (0.060)
Sex	Male	–	–
	Female	-0.184 (0.069)	-0.182 (0.105)
Ethnicity	White	–	–
	Black	0.120 (0.104)	0.209 (0.159)
Stage	1: Localised	–	–
	2: Regionalised	0.119 (0.073)	-0.190 (0.109)
	3: Distant	0.715 (0.111)	0.810 (0.186)

A.4. Sensitivity of results to period window choice

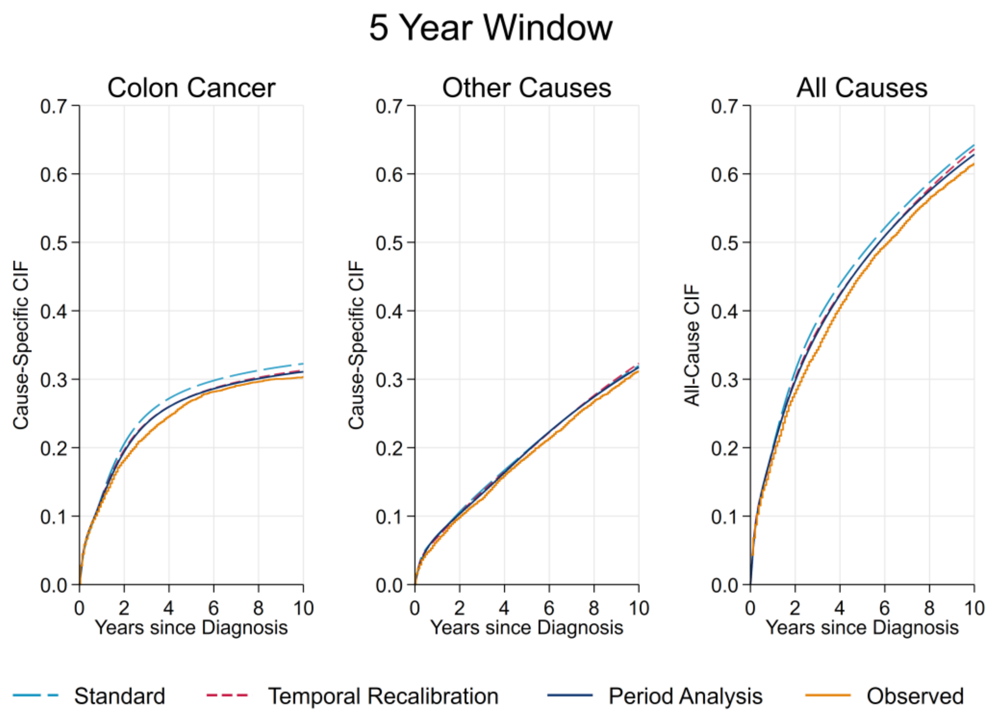

Figure 3: Comparison of the predicted and observed marginal cause-specific CIFs. The predictions for temporal recalibration and period analysis are from the models which used a 5 year window.

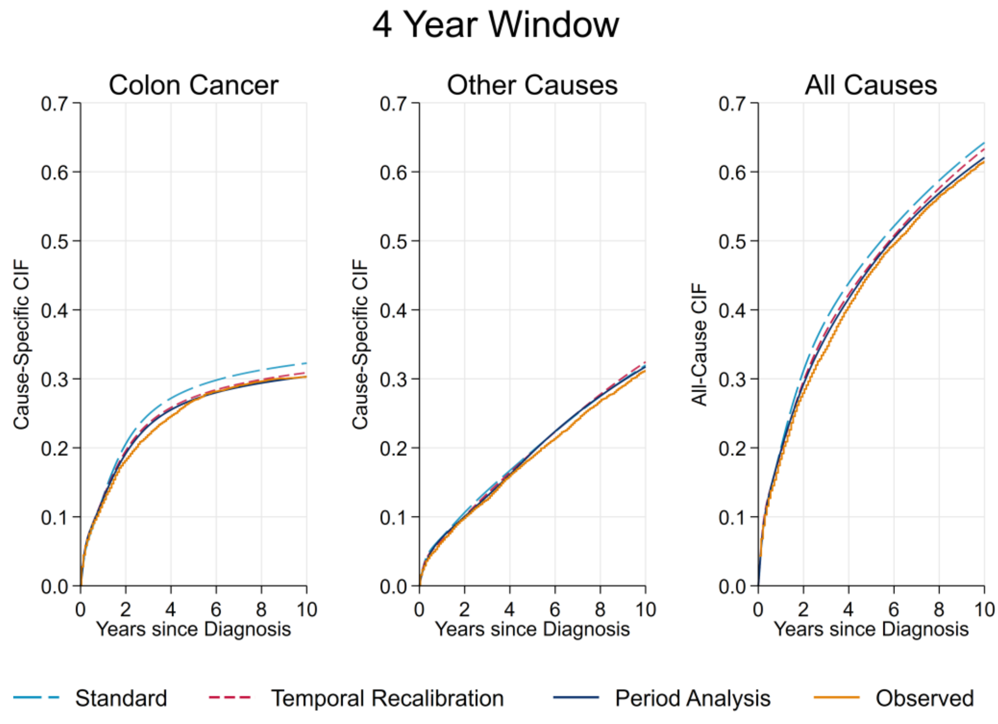

Figure 4: Comparison of the predicted and observed marginal cause-specific CIFs. The predictions for temporal recalibration and period analysis are from the models which used a 4 year window.

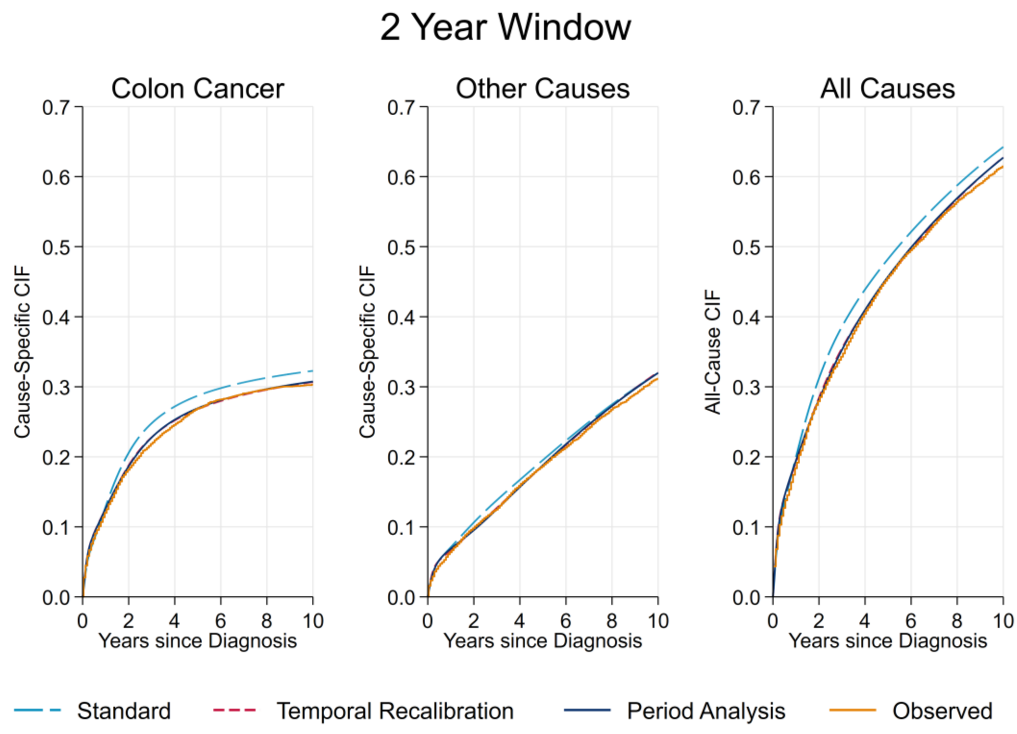

Figure 5: Comparison of the predicted and observed marginal cause-specific CIFs. The predictions for temporal recalibration and period analysis overlay almost exactly and are from the models which used a 2 year window.

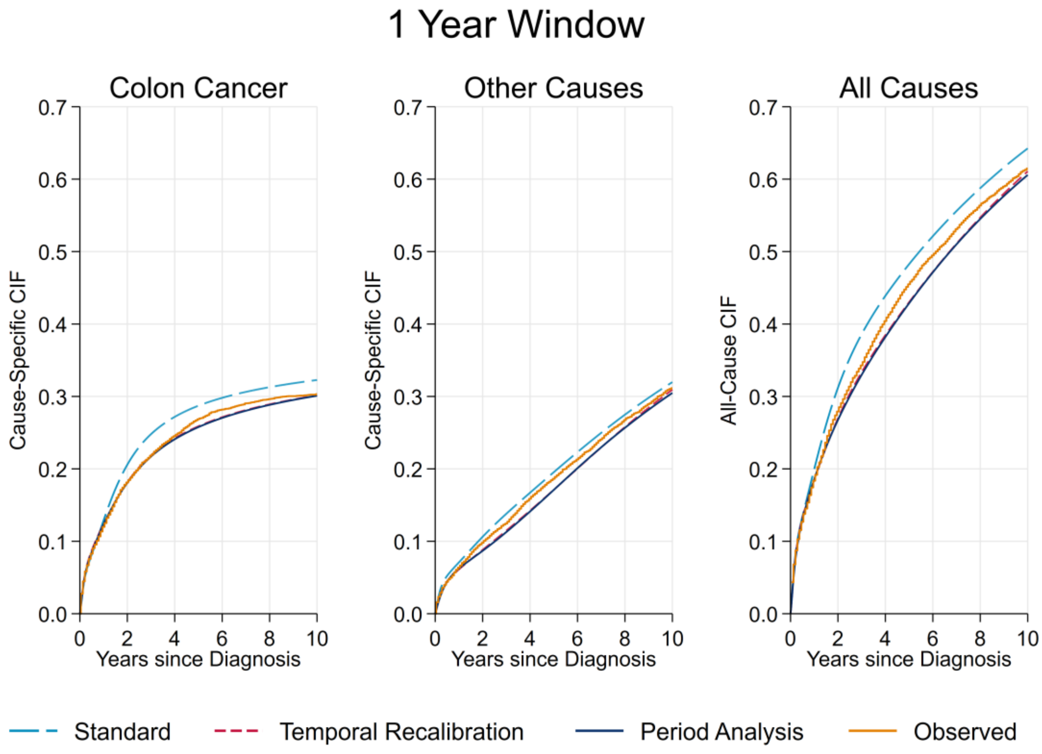

Figure 6: Comparison of the predicted and observed marginal cause-specific CIFs. The predictions for temporal recalibration and period analysis overlay almost exactly and are from the models which used a 1 year window.

A.5 Lung cancer

The analysis in the main paper was repeated for lung cancer. Both PH cause-specific hazard models included age (modelled with restricted cubic splines), sex, ethnicity and stage of tumour at diagnosis. Grade of tumour was also included in the model for death due to lung cancer. The models were developed using a 10% random sample of the patients diagnosed between 1995 and 2004 (follow-up until 31st December 2004) and the validation dataset comprised patients diagnosed the following year in 2005 (follow-up until 31st December 2015).

As shown in Table 4, there were around 4 times as many deaths due to cancer compared to other causes which may limit the choice of window for the other cause model. Using a narrow window, particularly for the other cause model, led to a larger degree of overfitting indicated by the decrease in the global shrinkage factor, see Table 5. Therefore, when using temporal recalibration and period analysis in this example, a 2 year window was used for the cancer model and a 4 year window was used for the other cause model.

Table 4: Sample size and number of events for each cause when using the standard approach or period analysis for model development

Method	Sample size	Diagnosed within the window	Deaths due to lung cancer	Deaths due to other causes
Standard approach	4,517	4,517	2,740	624
Period analysis: 5 year window	2,964	2,205	1,441	371
Period analysis: 4 year window	2,600	1,813	1,154	295
Period analysis: 3 year window	2,250	1,384	861	239
Period analysis: 2 year window	1,899	937	582	167
Period analysis: 1 year window	1,527	486	293	85

Table 5: Global shrinkage factor for each of the cause-specific models

Method	Lung Cancer	Other Causes
Standard approach	0.99	0.96
Period analysis: 5 year window	0.99	0.93
Period analysis: 4 year window	0.99	0.92
Period analysis: 3 year window	0.98	0.90
Period analysis: 2 year window	0.97	0.87
Period analysis: 1 year window	0.95	0.83

Using temporal recalibration or period analysis improved the calibration of the risk predictions due to lung cancer and the total risk of death for new patients by accounting for the improvement in survival over the development dataset, see Figure 7.

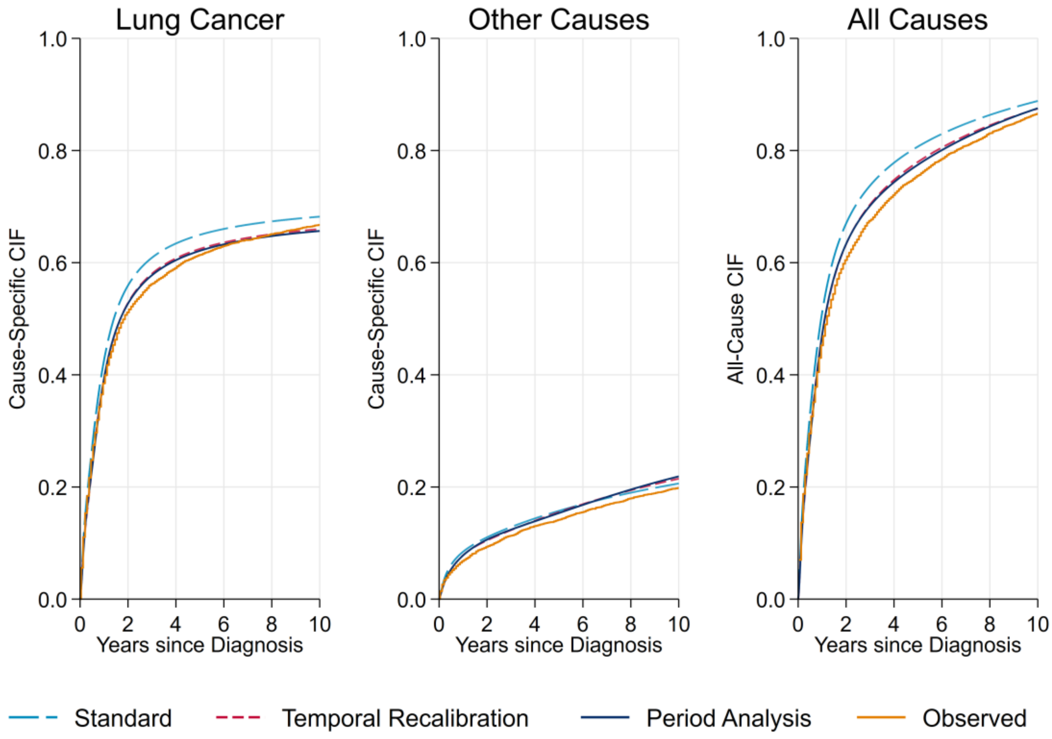

Figure 7: Comparison of the predicted and observed marginal cause-specific CIFs. The predictions for temporal recalibration and period analysis are from the models which used a 2 year window for the lung cancer model and a 4 year window for the other cause model.

Table 6: Difference between the predicted and observed cumulative incidence functions at 5 and 10 years post diagnosis. The predictions from using a 3 year window are presented for temporal recalibration and period analysis.

Time Point	Method	Colon Cancer	Other Causes	All Causes
5 years	Standard approach	0.036	0.016	0.052
	Temporal recalibration	0.011	0.015	0.026
	Period analysis	0.010	0.014	0.024
10 years	Standard approach	0.015	0.008	0.023
	Temporal recalibration	-0.001	0.019	0.010
	Period analysis	-0.001	0.021	0.012

A.6 Breast cancer

The analysis in the main paper was repeated for breast cancer. Both PH cause-specific hazard models included age (modelled with restricted cubic splines), ethnicity and stage of tumour at diagnosis. Grade of tumour was also included in the model for death due to breast cancer.

The models were developed using a 10% random sample of the patients diagnosed between 1995 and 2004 (follow-up until 31st December 2004) and the validation dataset comprised patients diagnosed the following year in 2005 (follow-up until 31st December 2015).

As shown in Table 7, there were approximately the same number of deaths due to cancer compared to other causes and so when using temporal recalibration and period analysis, a 3 year window was used for both cause-specific hazard models. In this example, even if a 2 year window were used, the global shrinkage factor remained high despite the large reduction in the number of events, see Table 8.

Table 7: Sample size and number of events for each cause when using the standard approach or period analysis for model development

Method	Sample size	Diagnosed within the window	Deaths due to breast cancer	Deaths due to other causes
Standard approach	9,243	9,243	904	805
Period analysis: 5 year window	8,764	4,950	635	600
Period analysis: 4 year window	8,537	3,997	504	509
Period analysis: 3 year window	8,301	3,052	388	397
Period analysis: 2 year window	8,060	2,037	262	283
Period analysis: 1 year window	7,786	1,083	135	149

Table 8: Difference between the predicted and observed cumulative incidence functions at 5 and 10 years post diagnosis. The predictions from using a 3 year window are presented for temporal recalibration and period analysis.

Method	Breast Cancer	Other Causes
Standard approach	0.99	0.99
Period analysis: 5 year window	0.99	0.99
Period analysis: 4 year window	0.99	0.99
Period analysis: 3 year window	0.98	0.99
Period analysis: 2 year window	0.97	0.98
Period analysis: 1 year window	0.94	0.96

Using temporal recalibration or period analysis improved the calibration of the risk predictions due to breast cancer and the total risk of death for new patients by accounting for the improvement in survival over the development dataset as shown in Figure 8.

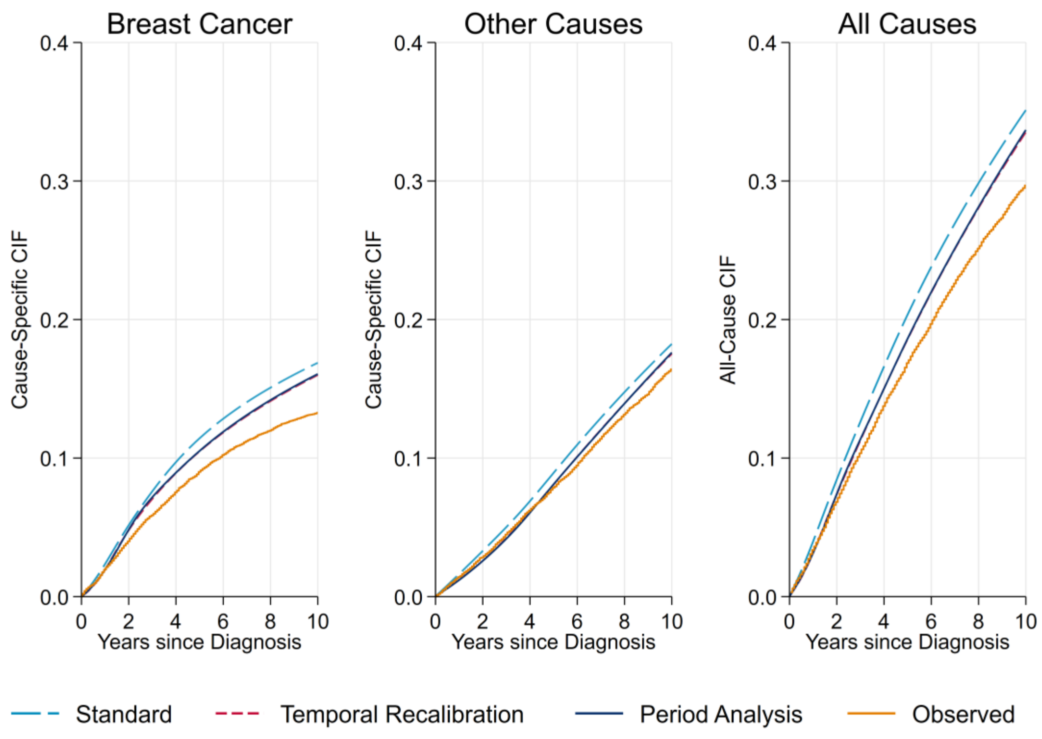

Figure 8: Comparison of the predicted and observed marginal cause-specific CIFs. The predictions for temporal recalibration and period analysis overlay almost exactly and are from the models which used a 3 year window.

Table 9: Difference between the predicted and observed cumulative incidence functions at 5 and 10 years post diagnosis. The predictions from using a 3 year window are presented for temporal recalibration and period analysis.

Time Point	Method	Colon Cancer	Other Causes	All Causes
5 years	Standard approach	0.024	0.011	0.035
	Temporal recalibration	0.015	0.002	0.017
	Period analysis	0.015	0.003	0.018
10 years	Standard approach	0.036	0.018	0.054
	Temporal recalibration	0.027	0.011	0.038
	Period analysis	0.028	0.012	0.040

A.7 Modelling on the subdistribution hazard scale

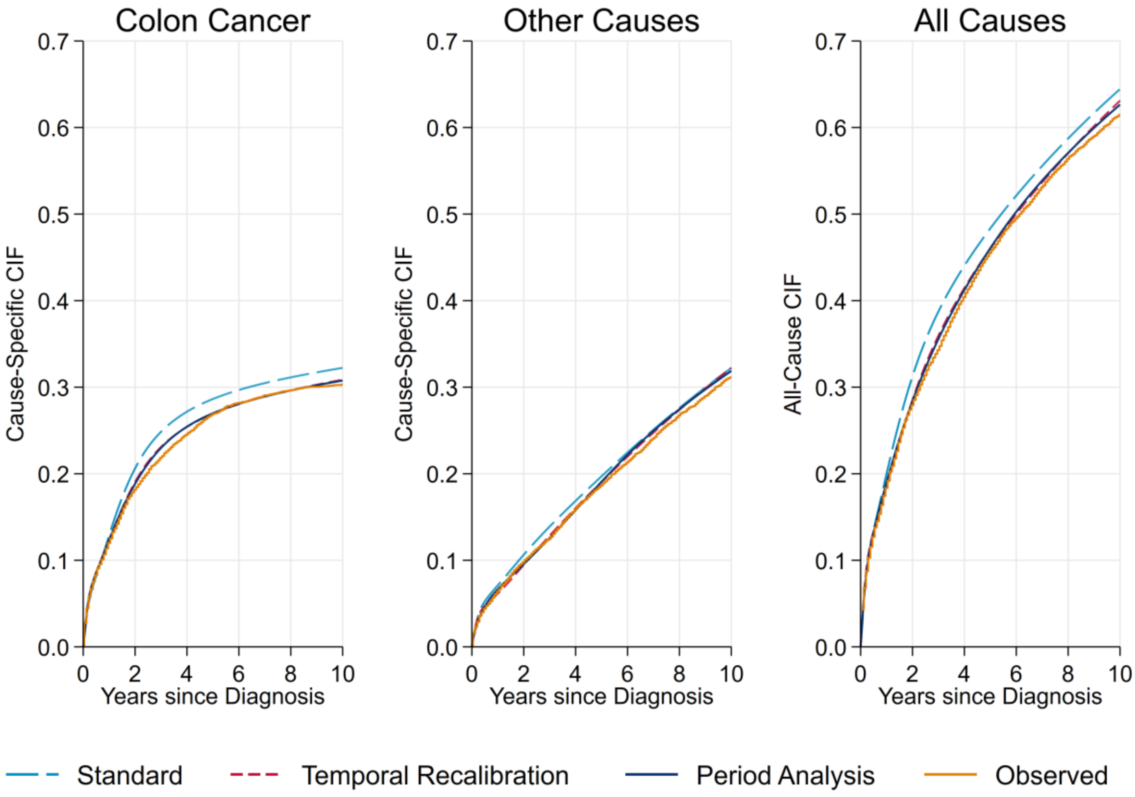

Figure 9: Comparison of the predicted and observed marginal cause-specific CIFs when using subdistribution hazard models for each cause model. The predictions for temporal recalibration and period analysis overlay almost exactly and are from the models which used a 3 year window.

Table 10: Difference between the predicted and observed cumulative incidence functions at 5 and 10 years post diagnosis. The predictions from using a 3 year window are presented for temporal recalibration and period analysis.

Time Point	Method	Colon Cancer	Other Causes	All Causes
5 years	Standard approach	0.018	0.011	0.029
	Temporal recalibration	0.001	0.004	0.006
	Period analysis	0.001	0.004	0.006
10 years	Standard approach	0.019	0.011	0.030
	Temporal recalibration	0.005	0.011	0.016
	Period analysis	0.004	0.007	0.012
